# Oxidation stability of biodiesel fuels and blends using the Rancimat and PetroOXY methods. Effect of 4-allyl-2,6-dimethoxyphenol and catechol as biodiesel additives on oxidation stability

**DOI:** 10.3389/fchem.2014.00043

**Published:** 2014-07-22

**Authors:** Lucía Botella, Fernando Bimbela, Lorena Martín, Jesús Arauzo, José L. Sánchez

**Affiliations:** ^1^Thermochemical Processes Research Group (GPT), Aragón Institute of Engineering Research (I3A), Universidad de ZaragozaZaragoza, Spain; ^2^Chemical and Environmental Engineering Department, Universidad de ZaragozaZaragoza, Spain

**Keywords:** biodiesel, oxidation stability, Rancimat, PetroOXY, additive, catechol, 4-allyl-2,6-dimethoxyphenol

## Abstract

In the present work, several fatty acid methyl esters (FAME) have been synthesized from various fatty acid feedstocks: used frying olive oil, pork fat, soybean, rapeseed, sunflower, and coconut. The oxidation stabilities of the biodiesel samples and of several blends have been measured simultaneously by both the Rancimat method, accepted by EN14112 standard, and the PetroOXY method, prEN16091 standard, with the aim of finding a correlation between both methodologies. Other biodiesel properties such as composition, cold filter plugging point (CFPP), flash point (FP), and kinematic viscosity have also been analyzed using standard methods in order to further characterize the biodiesel produced. In addition, the effect on the biodiesel properties of using 4-allyl-2,6-dimethoxyphenol and catechol as additives in biodiesel blends with rapeseed and with soybean has also been analyzed. The use of both antioxidants results in a considerable improvement in the oxidation stability of both types of biodiesel, especially using catechol. Adding catechol loads as low as 0.05% (m/m) in blends with soybean biodiesel and as low as 0.10% (m/m) in blends with rapeseed biodiesel is sufficient for the oxidation stabilities to comply with the restrictions established by the European EN14214 standard. An empirical linear equation is proposed to correlate the oxidation stability by the two methods, PetroOXY and Rancimat. It has been found that the presence of either catechol or 4-allyl-2,6-dimethoxyphenol as additives affects the correlation observed.

## Introduction

There are several reasons for the growing interest in biofuels including environmental concerns, climate change mitigation, ensuring secure energy supplies, and the development of cleaner, sustainable, and more environmentally friendly fuels. Besides, oil prices continue to rise steadily as a result of increased fossil fuel consumption (Gui et al., 2008) along with growing energy demands and needs. This in turn contributes to the worsening of already existing socioeconomic and environmental problems that need to be faced.

Industry is thus encouraged to continue to invest in R&D&I aimed at developing sustainable fuels from renewable sources with the highest quality standards. In particular, the development of sustainable, cost-competitive, and environmentally-friendly transportation fuels has led to a noticeable worldwide increase in the production and commercial use of biodiesel in the last decade. In addition to being a biodegradable and non-toxic fuel (Araújo et al., 2009; Sharma and Singh, 2009), biodiesel offers many other benefits over petroleum-derived fuels such as its lubricity (Muñoz et al., 2011), reduced exhaust emissions, being free of sulfur and aromatics (Kivevele et al., 2011), and the possibility of reducing our dependence on fossil energy sources, among others. Furthermore, biodiesel is completely miscible with petroleum diesel fuel (i.e., conventional diesel). Therefore, it can be used to produce biodiesel/conventional diesel blends.

Chemically, biodiesel is composed of a mixture of alkyl esters obtained in the transesterification of triglycerides from vegetable oils and animal fats, which is the conventional route for biodiesel production at an industrial scale. The triglycerides are reacted with a low molecular weight alcohol, usually methanol or ethanol, resulting in the formation of alkyl esters of fatty acids (i.e., biodiesel) and releasing glycerol as a by-product. The reaction is usually catalyzed with homogeneous catalysts, either acids or bases. Due to their wide availability at a reasonably economic cost, NaOH or KOH are typically employed in the industry (Lotero et al., 2005).

Given its biodegradable nature, biodiesel suffers from ageing, which poses one of its main advantages, environmentally speaking. On the other hand, this also implies that biodiesel is unstable and loses quality and properties over time. The oxidation stability of biodiesel is lower than that of petroleum-based diesel (Xin et al., 2009; Dantas et al., 2011; Jain and Sharma, 2011; Karavalakis et al., 2011; Kivevele et al., 2011), and this hinders its long-term storage.

Biodiesel must comply with the specifications established by relevant standards, such as EN 14214:2012 (EU) or ASTM D6751-12 (USA). Biodiesel/conventional diesel blends must also comply with a standard for automotive diesel (EN 590:2009+A1:2010). Among the quality parameters set by European and American standards, certain properties such as the oxidation stability of biodiesel or its behavior at low temperatures are of special importance and relevance. In both cases, the manufacture of quality biodiesel that matches the levels imposed by the standards for these properties usually poses a problem for producers, especially when using low cost and low quality raw materials. This inevitably leads to manufacturers adding expensive additives to improve their product in order to comply with the standards.

The present paper focuses on one of the most important biodiesel properties, the oxidation stability, and particularly on the methodologies used for its characterization. A review on biodiesel oxidation stability has recently been published by Pullen and Saeed (2012).

The mechanism for biodiesel oxidation is well known (Lapuerta et al., 2012). The oxidation stability depends on the composition of the oil source, the unsaturated and polyunsaturated methyl esters being the more reactive species. The greater the level of unsaturation in an alkyl ester, the more susceptible it will be to oxidation. In any case, besides depending on the degree and configurations of olefinic unsaturations, the resistance of biodiesel against oxidation will also be dependent on the presence of antioxidants and on storage conditions.

The oxidation kinetics of biodiesel have been studied as well as the effect of antioxidants (Xin et al., 2009). Since oxidation products can cause many problems in diesel engines (Knothe, 2007; Lapuerta et al., 2008), it is important to prevent oxidation by adding additives such as butylated hydroxyanisole (BHA), pyrogallol (PA), butylated hydroxytoluene (BHT), and other commercial products such as Baynox (Lanxess), Bioextend (Eastman), and Ethanox 4760E (Albemarle). Several papers dealing with natural (Liang et al., 2006; Santos et al., 2012) and synthetic (Dunn, 2005; Liang et al., 2006; Karavalakis et al., 2011; Focke et al., 2012) antioxidants for biodiesel can be found in the literature, reflecting that the search for new alternative low-cost additives is currently a major issue.

In this context, the use of different experimental techniques to evaluate the oxidation stability of biodiesel has been discussed (Hoshino et al., 2007), as methods based on FTIR (Araújo et al., 2011), thermogravimetry (Kivevele et al., 2011; Santos et al., 2012), or DSC (García-Pérez et al., 2010). The standardized accelerated oxidation test accepted in EN14112 and ASTM D6751 standards is the usually called Rancimat method (Maia et al., 2011; Focke et al., 2012; Lapuerta et al., 2012). A new determination method was reported by Neumann et al. (2008), which provides a more rapid test to measure oxidation stability than the Rancimat method, called PetroOXY method and it is described in the prEN16091 standard.

The European standard for biodiesel (EN 14112) sets a lower limit of 8 h as the minimum induction period, while 3 h of oxidation stability is established by the standard ASTM D6751-12, both determined by using the Rancimat method. It must be considered that although both standards are for pure biodiesel (B100), the ASTM is intended for biodiesel to be used in blends with fossil diesel.

An important advantage of the PetroOXY method over the Rancimat method is the significantly shorter duration of that test for determining the oxidation stability of a given sample. This is very important when testing and developing new additives for fuels. The PetroOXY test is standardized in the USA (ASTM D7545-14) but to the best of our knowledge it has not been accepted in Europe, and a minimum value of induction time using the PetroOXY test has yet to be accepted in European standards.

Although both methods are designed for determining the oxidation stability of biodiesel, each method is based on the measurement of different properties (Ramalho et al., 2011). The Rancimat method provides an incomplete analysis of the oxidation stability of the sample because only the highly volatile oxidation products are detected through a combination of distillation and conductivity, whereas the PetroOXY method includes all volatile and non-volatile oxidation products. Therefore, unlike the Rancimat method, the PetroOXY method provides a complete analysis of the sample's oxidation stability through the measurement of the induction period related to the pressure loss in an oxygen atmosphere (Neumann et al., 2008).

While many papers dealing with the evaluation of biodiesel oxidation stability using the Rancimat method can be found in the literature, only a few deal with the use of the PetroOXY method. Neumann et al. (2008) and Araújo et al. (2009) found linear correlations between the Rancimat and PetroOXY methods. In Neumann et al. (2008), soybean oil biodiesel was blended with conventional diesel in different proportions (B2 through B100) and a linear correlation between both methods was found in all the range tested. In Araújo et al. (2009), four different additives were tested using castor oil biodiesel at additive concentrations up to 5880 ppm, a linear relationship between the two methods for antioxidant concentrations between zero and 3000 ppm is reported. In these works, the PetroOXY method proved to have good repeatability (less than 5% error) and good reproducibility (less than 8% error) between the different replicates. Moreover, Damasceno et al. (2013) investigated the effect of three different antioxidants (TBHQ, caffeic acid, and ferulic acid), with concentrations of 1000 ppm, on the oxidation stability of ethyl soybean biodiesel using both methods. The study aimed at comparing the stability performance of the three additives. The authors reported differences in the oxidation stability measurements using both instruments that were attributed to measuring different oxidation states.

In this context, the main goal of this paper is to establish whether a correlation between both methods of oxidation measurement exists, regardless of the raw material employed for producing the biodiesel or the presence of additives that could modify the oxidation stability.

To carry out the study, several fatty acid methyl esters (FAME) were synthesized from different fatty acid stocks and their oxidation stabilities were measured simultaneously by both methods (Rancimat and PetroOXY). In addition, the effect on the oxidation stability of blending biodiesel with additives has also been analyzed. Other biodiesel properties such as the composition, the cold filter plugging point (CFPP), the flash point (FP), and kinematic viscosity were measured using standard methods in order to further characterize the biodiesel produced.

## Materials and methods

### Materials and biodiesel preparation

The present work covers a wide range of biodiesel compositions. Up to 12 different biodiesel samples were prepared, six of them from six different raw materials and the other six consisting of binary blends. To the best of our knowledge, there are no other studies in the literature that have measured oxidation stabilities for as long as 800 min of as many heterogeneous and different samples as in this work.

The following raw materials were selected for producing biodiesel: refined sunflower oil (Hacendado, Spain), refined rapeseed oil (Ja!, Germany), refined soybean oil (Sojola, Germany), pure coconut oil (KTC, Sri Lanka), used domestic frying oil (mostly olive oil), and commercial edible pork fat (Grup Roma-Avinyó, Spain). As mentioned, biodiesel produced from these raw materials was also blended in different proportions of product pairs. The blends that were studied are: pork fat/used frying olive oil (UFOO), soybean/rapeseed, and sunflower/coconut. Two different mass ratios were selected for all the blends prepared: 30/70 and 70/30 (expressed as relative percentage mass ratios). Using these raw materials and blends resulted in a wide range of different biodiesel compositions and, subsequently, different oxidation stability values.

Reagents used during the biodiesel synthesis and purification steps were: methanol (assay (GLC) = 99.9%, Carlo Erba Reagents), sulfuric acid (95%, Fisher Chemical), potassium hydroxide (assay >85%, Carlo Erba reagents), and magnesium sulfate (anhydrous, Scharlau).

Two phenolic antioxidants were used for doping the different biodiesels. Catechol (CAS number 120-80-9, assay >99%, Fisher Chemical) and 4-allyl-2,6-dimethoxyphenol (CAS number 6627-88-9, assay = 90%, Sigma-Aldrich) were selected as biodiesel additives. Catechol is typically found in the formulations of some commercial antioxidant additives for biodiesel (Jain and Sharma, 2010), whereas 4-allyl-2,6-dimethoxyphenol is a phenolic compound that can be typically found in biomass pyrolysis liquids (García-Pérez et al., 2007a,b) and thus can constitute a renewable additive. The effect of these two compounds was investigated separately by blending them in small amounts [0.05, 0.1, and 0.3% (m/m)] with biodiesel produced from two of the selected raw materials in this study, soybean and rapeseed.

A solution of methyl heptadecanoate (standard for GC, Fluka Analytical) in heptane (standard for GC, Fluka Analytical) was used as the internal standard in the quantitative analyses of the biodiesel products carried out by gas chromatography with a flame ionization detector (GC-FID). A Supelco™ standard 37 Component FAME Mix with 37 components was used in order to identify the different methyl esters in the chromatograms.

Biodiesel was produced with methanol as a reagent by alkaline transesterification in a batch reactor (2 dm^3^ glass vessel) equipped with a mechanical stirrer, a condenser and a thermocouple. The methanolysis reaction temperature was set at 60°C and the relative centrifugal force (RCF) was set at 1 in order to avoid mass transfer problems. A complete description of the experimental procedure can be found elsewhere (García et al., 2011).

### Raw materials and biodiesel characterization

Fatty acid composition of the oils and pork fat was determined by GC-FID in accordance with EN 14103 (2003) and ISO 5508 (1990). Biodiesel methyl esters were quantified by GC-FID using an external standard. The analyses were done in an Agilent 6890 Series GC System gas chromatograph, with a DB-225ms (Agilent) column (30 m × 0.25 mm × 0.25 μm). The injector and detector (FID) temperatures were set at 250°C. Helium was used as carrier gas with a flow of 1 cm^3^/min. Injection was made in a split mode, using a split flow ratio of 35:1; the volume injected was 10^−9^ m^3^. The following temperature program was used: an initial temperature of 170°C, followed by heating at 3°C/min to 203°C, at 1.5°C/min to 214°C, and then at 5°C/min to 230°C. This temperature was maintained for 16 min. Using this method, FAME could be separated, identified, and quantified based on their selective retention according to their polarities and by comparison with the retention times and response factors obtained for the standard mix.

Oxidation stability was measured using PetroOXY (Petrotest) equipment following the method described in prEN 16091:2010. 5 cm^3^ of the sample are placed in the reaction vessel, which is pressurized with oxygen at 700 kPa and heated to 140°C. The oxygen is consumed during the oxidation and the subsequent pressure drop is recorded every second with a data acquisition system. The elapsed time from the start to the breakpoint is the induction period at the test temperature of 140°C. In addition, the induction times of the same samples were measured by the Rancimat method in an external laboratory (BioArag S.L.) in accordance with the standard EN 14112. Both analyses, Rancimat and PetroOXY, were carried out at the same time for each sample to avoid any variation in the degrees of oxidation.

The CFPPs were measured with a FPP 5GS instrument (ISL), based on the standard EN 116. A specific volume of the sample (45 cm^3^) is steadily cooled down and passed through a 45 μm mesh filter under vacuum (1.961 kPa). Paraffin crystals become solidified and deposited on the filter as a result of the low temperature, eventually clogging the filter. The CFPP is determined when the sample ceases to flow through the filter within 60 s or fails to return.

FP determinations using a Pensky-Martens closed cup tester were carried out in a PMA4 (Petrotest) in accordance with ASTM D93. The temperature of the sample is raised and a small heat flux is periodically applied to the sample surface by means of an electric resistance. The sample becomes ignited when the FP temperature is reached, which is recorded by the instrument.

Kinematic viscosity values were determined with a Cannon-Fenske viscometer (Cannon Instrument Co., model 150 T845) at 40°C following the standard method EN ISO 3104. The elapsed time for a fixed sample volume to flow between two marks in the instrument is recorded and the viscosity is calculated using the viscosimeter constant provided by the manufacturer.

## Results and discussion

### Characterization of the different biodiesel and blends produced

The first set of samples was produced and directly measured without adding any additives. Tables 1, 2 show the fatty acid semiquantitive analyses determined by GC/FID for all the biodiesel samples and blends, respectively. As shown in Table 1, the selected vegetable oils and animal fats have very different compositions, in order to study the correlation between methods over a wide range of compositions. Compositional parameters pertaining to the initial fatty oil or ester include the ester content, the fatty acid chain distribution within the fatty oil or ester, and the type and extent of olefinic unsaturation. For this reason, the degree of total unsaturation of each sample appears in Tables 1, 2, calculated as the sum of the contribution of each of the unsaturated methyl esters that contain biodiesel. As shown, there are different types of biodiesel high in unsaturated esters, such as soybean, rapeseed, sunflower, used frying olive oil biodiesel, and mixtures thereof, and others with a low content of unsaturated esters, such as coconut and animal fat biodiesel and mixtures thereof.

**Table 1:** **Quantitative analyses by GC/FID of the biodiesel produced from pure raw materials [% (m/m)]**.

**Sample Biodiesel**	**B1 UFOO^a^**	**B2 Pork fat**	**B3 Soybean**	**B4 Rapeseed**	**B5 Sunflower**	**B6 Coconut**
Caprylic (C8:0)						7.8
Capric (C10:0)						6.6
Lauric (C12:0)						48.2
Myristic (C14:0)		1.6				17.3
Palmitic (C16:0)	12.9	27.0	10.3	4.6	6.6	9.2
Margaric (C17:0)		0.4				
Stearic (C18:0)	2.8	17.0	3.0	1.6	3.5	2.7
Arachidic (C20:0)				0.6		
Lignoceric (C24:0)			0.5		0.7	
Palmitoleic (C16:1)	1.1	1.7				
Oleic (C18:1)	72.2	35.3	28.5	64.2	27.7	6.6
Gadoleic (C20:1)		0.6		1.3		
Linoleic (C18:2)	10.5	15.0	52.8	19.3	61.6	1.8
Linolenic (C18:3)	0.6	1.1	4.9	8.4		
Unsaturation degree	84.3	53.6	86.2	93.3	89.3	8.5
Ratio poly unsaturated/mono unsaturated	0.2	0.4	2.0	0.4	2.2	0.3

a Used frying olive oil.

**Table 2:** **Quantitative analyses by GC/FID of the biodiesel binary blends produced [(% (m/m)]**.

**Sample Raw material 1 Raw material 2 wt. ratio 1:2**	**B7 Pork fat UFOO^a^ 70:30**	**B8 Pork fat UFOO^a^ 30:70**	**B9 Soybean Rapeseed 70:30**	**B10 Soybean Rapeseed 30:70**	**B11 Sunflower Coconut 70:30**	**B12 Sunflower Coconut 30:70**
Caprylic (C8:0)					2.3	5.7
Capric (C10:0)					2.0	4.8
Lauric (C12:0)					14.8	35.4
Myristic (C14:0)	1.2	0.6			5.4	12.6
Palmitic (C16:0)	22.7	17.5	9.0	5.9	7.3	8.3
Margaric (C17:0)	0.4					
Stearic (C18:0)	12.7	7.1	2.6	2.1	3.3	2.9
Arachidic (C20:0)				0.5		
Lignoceric (C24:0)				0.3	0.4	
Palmitoleic (C16:1)	1.5	1.3				
Oleic (C18:1)	46.4	61.7	39.1	53.7	21.3	11.8
Gadoleic (C20:1)	0.5		0.5	1.0		
Linoleic (C18:2)	13.6	11.2	42.8	29.1	43.2	18.5
Linolenic (C18:3)	0.9	0.7	5.9	7.4		
Unsaturation degree	62.8	74.8	88.4	91.2	64.6	30.4
Ratio poly unsaturated/mono unsaturated	0.3	0.2	1.2	0.7	2.0	1.6

a Used frying olive oil.

The results of different physicochemical properties measured in the different biodiesels and biodiesel blends are shown in Table 3. The measured properties were oxidation stability, determined by both the Rancimat and PetroOXY methods, CFPP, FP, and kinematic viscosity.

**Table 3:** **Physicochemical properties of the biodiesel and biodiesel blends produced**.

**Sample**	**PetroOXY (min)**	**Rancimat (min)**	**CFPP (°C)**	**FP (°C)**	**Viscosity (m^2^/s·**10**^6^)^a^**
B1	31.4	864	−2.9	178	4.6
B2	14.6	330	11.4	172.5	4.6
B3	15.5	231	−6.2	172.5	4.3
B4	19.0	310	−10.6	182	4.6
B5	6.8	26	−4	139	4.3
B6	29.3	770	−7.3	109	2.8
B7	20.0	478	8.1	174.5	4.7
B8	27.1	657	9.1	178	5.0
B9	18.4	257	−9.5	172.5	4.4
B10	18.1	244	−8.4	158	4.4
B11	9.0	49	−8.4	139	3.7
B12	12.0	174	−8.4	119	3.2

a Measured at 40°C.

All the biodiesel samples had low viscosities at 40°C, around 3–5·10^−6^ m^2^/s. They all had FP values above 101°C, which is good in terms of transportation safety issues. The highest value, 182°C, was obtained for the rapeseed biodiesel. The UFOO, pork fat and soybean biodiesel samples had similar FP values, very close to that of the rapeseed biodiesel, in the range of 172.5–178°C. The sunflower and coconut had the lowest FPs, 139 and 109°C, respectively. No significant changes in the FP values were observed in the samples prepared with mixtures of raw materials.

The lowest CFPP values were obtained for the rapeseed biodiesel (−10.6°C) and the highest for the pork fat (11.4°C). The biodiesel samples elaborated with the rest of the pure raw materials presented CFPP values of between −3 and −7°C approximately. With regard to the biodiesel samples elaborated using mixed raw materials, the CFPP obtained are between the values obtained for the pure raw materials. As it could be expected, the highest values were obtained for the pork fat/UFOO biodiesel samples, over 8°C.

The effect of two additives on the oxidation stability has also been studied. Two batches of soybean and rapeseed biodiesel were prepared and different blends with additives were produced from these batches. Table 4 presents the results obtained for the biodiesel samples produced from soybean and rapeseed and their blends with different contents of the selected additives, 4-allyl-2,6-dimethoxyphenol (A) and catechol (C). Both additives exert an effect on the oxidation stabilities of soybean and rapeseed biodiesel. Regarding the use of the two additives, it is worth mentioning that, despite the very low amounts used, the addition of these compounds to both biodiesel samples significantly enhances the oxidation stability, especially in the case of catechol. The influence of these two additives will be further discussed in section Influence of Additives. Regarding the rest of the properties measured, although there are not enough data to carry out a proper analysis, the only difference seems to be caused in FP by the use of additive A with soybean biodiesel.

**Table 4:** **Physicochemical properties of the biodiesel blends with additives: Results for 4-allyl-2,6-dimethoxyphenol (A) and catechol (C), blended with soybean and rapeseed biodiesel**.

**Sample**	**Raw material/additive**	**Additive content [% (m/m)]**	**PetroOXY (min)**	**Rancimat (min)**	**CFPP (°C)**	**FP (°C)**	**Viscosity (m^2^/s·**10**^6^)^a^**
B13	Soybean/-	0	7.08	151.2	−6.2	176.5	4.2
BA1	Soybean/A	0.05	11.33	186.6	−5.1	99	4.23
BA2	Soybean/A	0.1	16.48	283.8	−6.2	99	4.19
BA3	Soybeam/A	0.3	23.62	323.7	−6.2	164.5	4.21
BC1	Soybeam/C	0.05	34.72	562.8	−5.1	176.5	4.17
BC2	Soybean/C	0.1	46.88	687	−5.1	176.5	4.19
BC3	Soybean/C	0.3	55.39	711.6	−5.1	170.5	4.21
B14	Rapeseed/-	0	14.93	208.2	−9.5	179	4.52
BA4	Rapeseed/A	0.05	20.2	349.2	−11.7	128.5	4.57
BA5	Rapeseed/A	0.1	24.28	447.6	−12.8	172.5	4.59
BA6	Rapeseed/A	0.3	36.75	497.7	−12.8	176.5	4.52
BC4	Rapeseed/C	0.05	29.83	447.6	−11.7	162.5	4.51
BC5	Rapeseed/C	0.1	45.73	570.4	−11.7	158.5	4.51
BC6	Rapeseed/C	0.3	52.63	673.2	−13.9	174.5	4.55

a Measured at 40°C.

### Preliminary study on long-term measurements of oxidation stability

**Figure 1:**
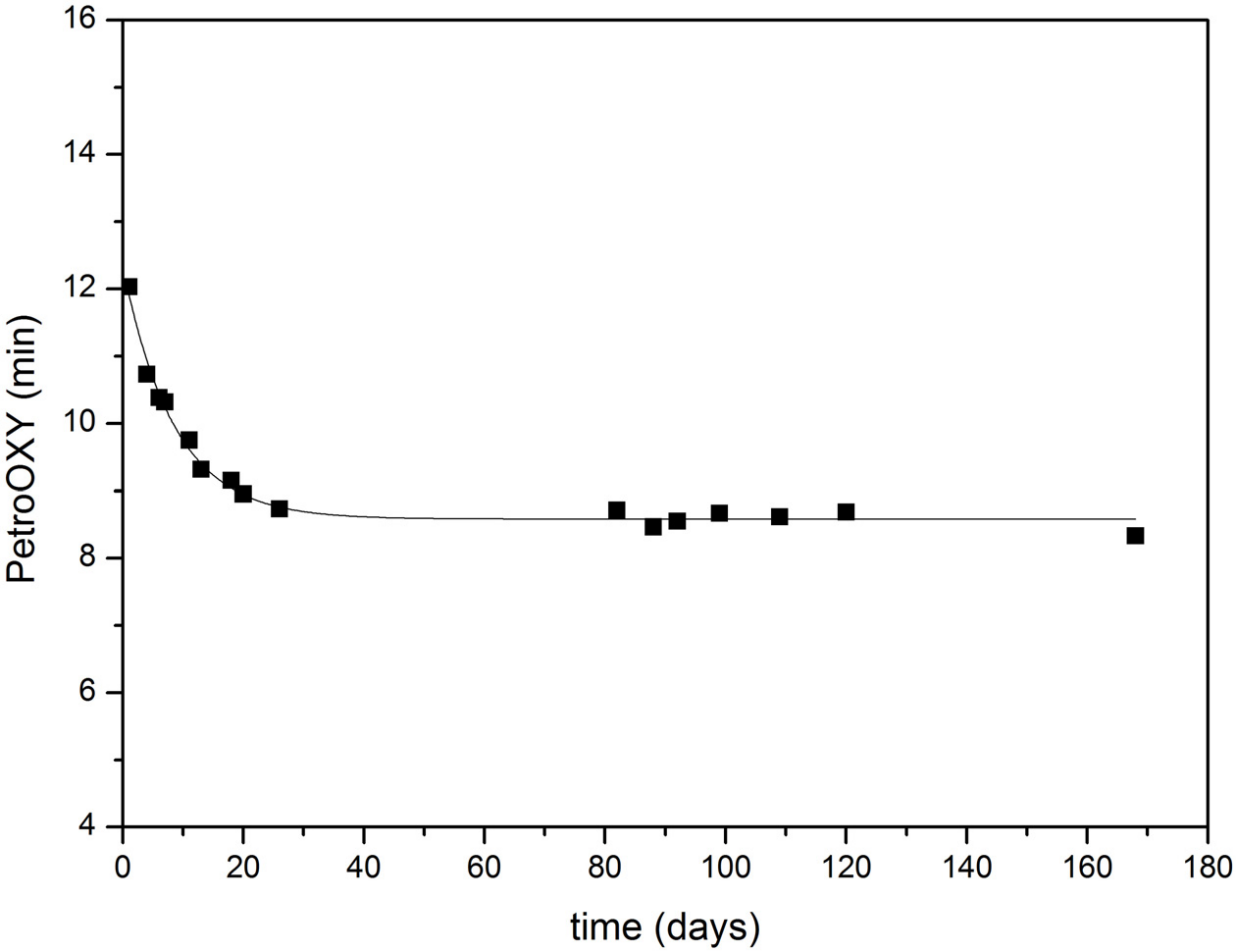
**Evolution of the oxidation stability of sunflower oil biodiesel over time determined with the PetroOXY method**. (■ biodiesel samples).

In order to find a correlation between the Rancimat and the PetroOXY methods, it is important to measure simultaneously the oxidation stability of the samples in both systems. This is because the induction time changes rapidly in the first hours after biodiesel has been prepared due to the high rate of the oxidation reaction. In order to gain an insight into the typical induction times obtained using the PetroOXY method, a preliminary series of oxidation stability measurements were carried out with a sunflower oil biodiesel (sample B5, Table 1) during more than 150 days. All the test samples were stored under identical conditions (in an opaque tank under atmospheric pressure and at 25°C). Figure 1 shows the evolution of the oxidation stability with time. The sunflower biodiesel produced immediately after the final cleaning step of the biodiesel synthesis had an initial oxidation stability of 12 min. During the first few days the oxidation stability followed an exponential decay over time down to PetroOXY induction time values of around 8.6 min. This represents the oxidation stability of the biodiesel in long-term storage. The experimental data have been fitted to an exponential equation, where t is the time in days (Equation 1). The regression coefficient is *R*^2^ = 0.993, which indicates a good fit.

Given the methodology followed in the PetroOXY oxidation stability test, in which the oxidation stability is determined as the time required so as not to have a decrease in the oxygen pressure greater than 10% in the instrument, the data obtained using this methodology is directly related to the concentration of species that can be oxidized. This implies that the kinetics of the biodiesel oxidation reaction correspond to a first order reaction, in agreement with the literature (Xin et al., 2009; Machado et al., 2013).

(1) $$\text{PetroOXY }\left(\min\right)\;=\;8.583\;+\;3.759\;\cdot \;{\text{e}}^{\left(-0.117\text{t}\right)}$$

### Correlation rancimat—petroOXY oxidation stability tests

The measurements of the oxidation stabilities of the samples reveals the significant differences that can be found depending on the raw material selected for producing the biodiesel. From the results in Table 3 it can be observed that samples B1 and B6, used frying olive oil and coconut biodiesel, respectively, show the highest oxidation stability, whereas sunflower biodiesel has the lowest. Pork fat, soybean, rapeseed, and the blends of soybean and rapeseed show intermediate values. The different blends prepared have intermediate oxidation stability values, comprised between those of the corresponding biodiesel samples from the pure raw materials.

This indicates a direct relationship of the raw material composition with the oxidation stability of the biodiesel produced (McCormick et al., 2007; Ramos et al., 2009), particularly with regard to the contents in certain unsaturated fatty acids (linolenic, linoleic, and oleic). Thus, the raw materials having the lowest contents of oleic acid and the greatest contents of linoleic and linolenic acid, i.e., a greater proportion of compounds with multiple unsaturation as against compounds with a single unsaturation, had the lowest oxidation stabilities. This is particularly noteworthy in the case of used frying olive oil, which has the lowest poly-unsaturated/mono-unsaturated ratio (Table 1). These facts justify the need for seeking additives that might inhibit the oxidation reactions caused by the presence of unsaturated compounds in biodiesel.

**Figure 2:**
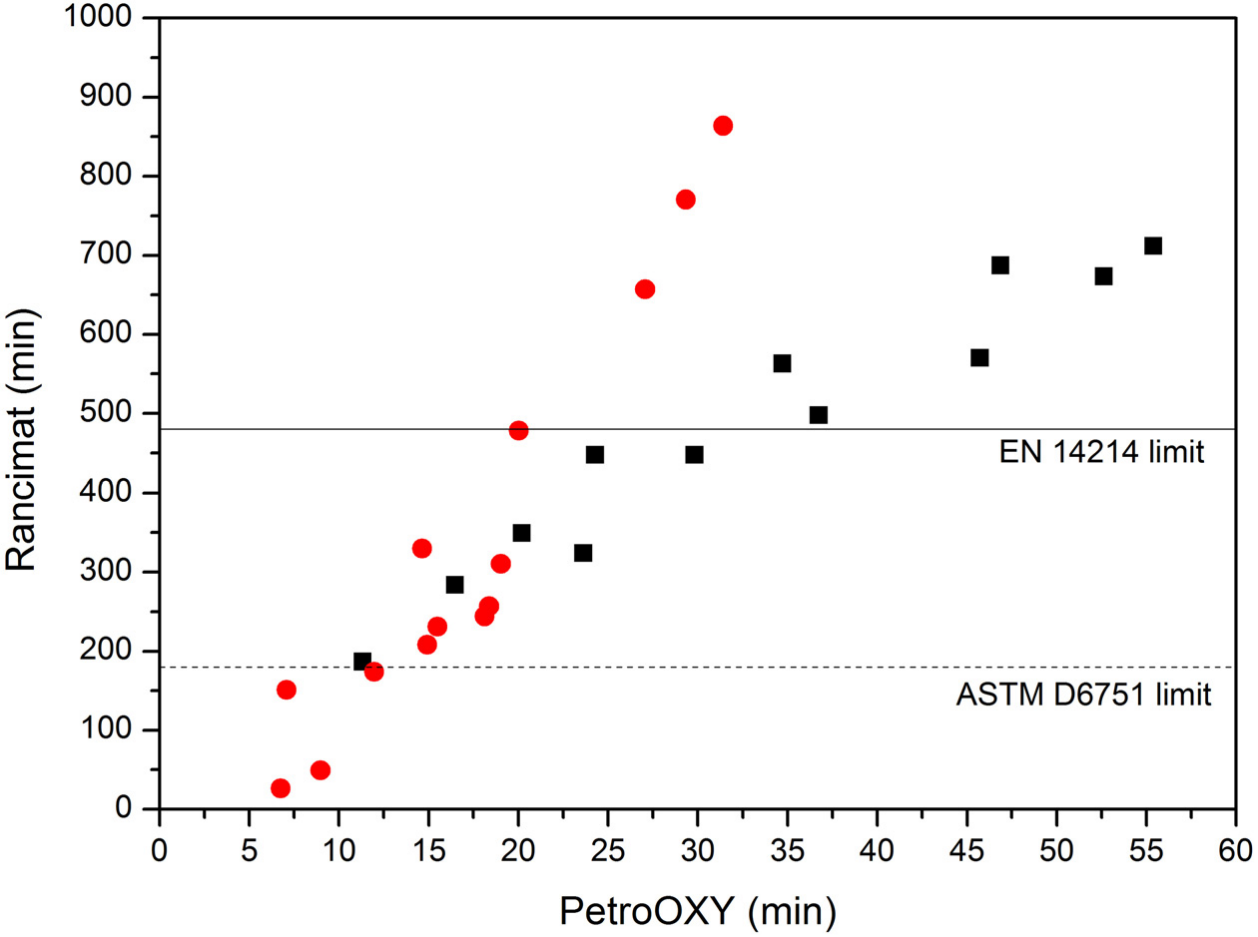
**Rancimat vs. PetroOXY oxidation stability measurements of the different biodiesel samples prepared (■ biodiesel samples without additive, 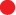 soy and rapeseed biodiesel with additive)**.

Figure 2 shows the oxidation stability values measured with the PetroOXY method vs. the values obtained in the Rancimat apparatus. The limits imposed by the EN 14214 and the ASTM D6751 standards for oxidation stability (480 and 180 min, respectively) are also shown. It can be observed that, according to the American standard, most of the biodiesel samples produced without additive would be valid, except for samples B5 (sunflower biodiesel) and B11 [sunflower/coconut (70:30)]. Sample B12, sunflower/coconut (30:70), is very close to the threshold value imposed by the ASTM D6751 standard. Hence, it could be concluded that sunflower, either pure or blended, would not be a suitable raw material for producing biodiesel even considering the less strict standard, unless antioxidant additives are used in the formulation of the final product.

The limits imposed by standard EN 14214 are stricter. Only samples B1 (UFOO), B6 (coconut), and B8 [Pork fat/UFOO (30:70)] fulfill the oxidation stability limit set by EN 14214 without needing further antioxidant additives in the formulation.

In order to propose a correlation between the oxidation time measured with the PetroOXY and the Rancimat induction times, the whole set of 26 data shown in Tables 3, 4 have been used in the regression study. The R free software program has been used for data fitting (R Core Team., 2012). In the analyses carried out, it has been considered that the linear correlation between the values obtained with the PetroOXY and the Rancimat tests can be affected by different factors, such as biodiesel composition, additive used and amount, or presence or not of additive. In order to discriminate between the models, an ANOVA test based on the F distribution has been applied, using a 95% of confidence.

It can be observed in Figure 2 that, for a similar time obtained using the PetroOXY method, the Rancimat induction time is higher for the biodiesel samples without additive. This observation is confirmed after the statistical analyses, where different possible linear relations between the variables have been tested. The conclusions obtained from these analyses using the available data can be summarized as follows:

- There are not significant differences in the linear correlation between PetroOXY and Rancimat times regarding the composition of the biodiesel used. It must be taken into account that the number of mixtures tested and raw materials is limited in this study. Hence, the simultaneous measurement of this property by the two methods from a higher number of raw materials and proportions could be desirable.
- There are not any significant differences in the correlation either regarding the two compounds used as additives (catechol and 4-allyl-2,6-dimethoxyphenol) or the amount used (0.05–0.3% mass fraction).
- There is a statistically significant difference in the regression regarding both the intercept and the slope of the linear regression of PetroOXY vs. Rancimat oxidation times between the data sets obtained with or without additive.

Thus, the best regression model is shown in Equation 2:

(2) $$\begin{array}{l} \text{Rancimat }(\min)\;=\;(31.89-20.63\cdot \text{f})\;\cdot \text{ PetroOXY }(\min)\\ \;+\;(-214.65\;+\;319.68\;\cdot \text{ f}) \end{array}$$

Where f equals 0 if no additive is used and 1 if an additive has been mixed with biodiesel. The adjusted coefficient of determination, *R*^2^, obtained is 0.915. It can thus be concluded that there is not a single linear relationship between the measurements of the two instruments. Nonetheless, the correlation proposed can be useful in order to get a quick determination of the oxidation stability using the PetroOXY method.

**Figure 3:**
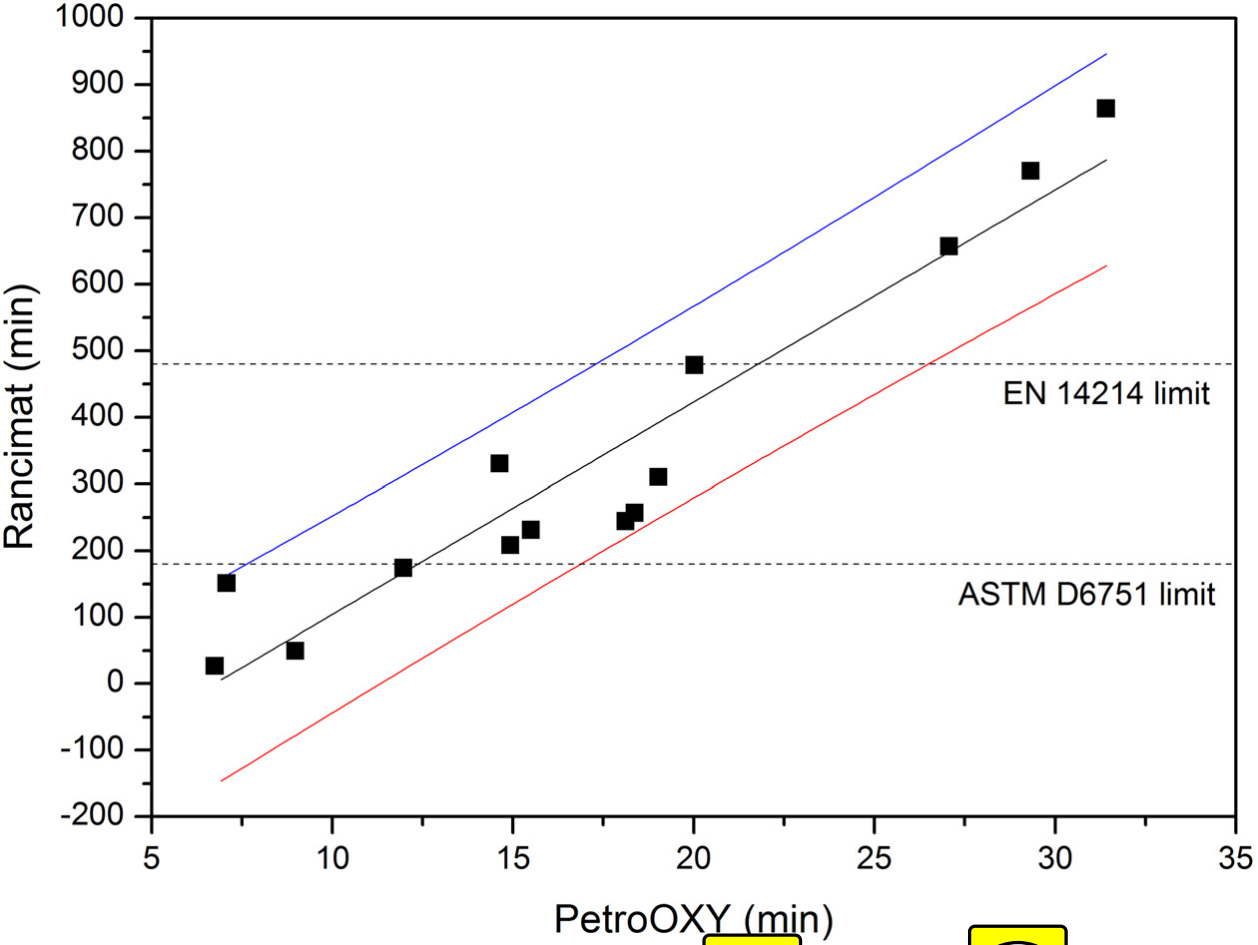
**Regression line (−), upper (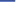), and lower (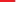) prediction intervals and experimental data (■)**. Biodiesel without additive.

**Figure 4:**
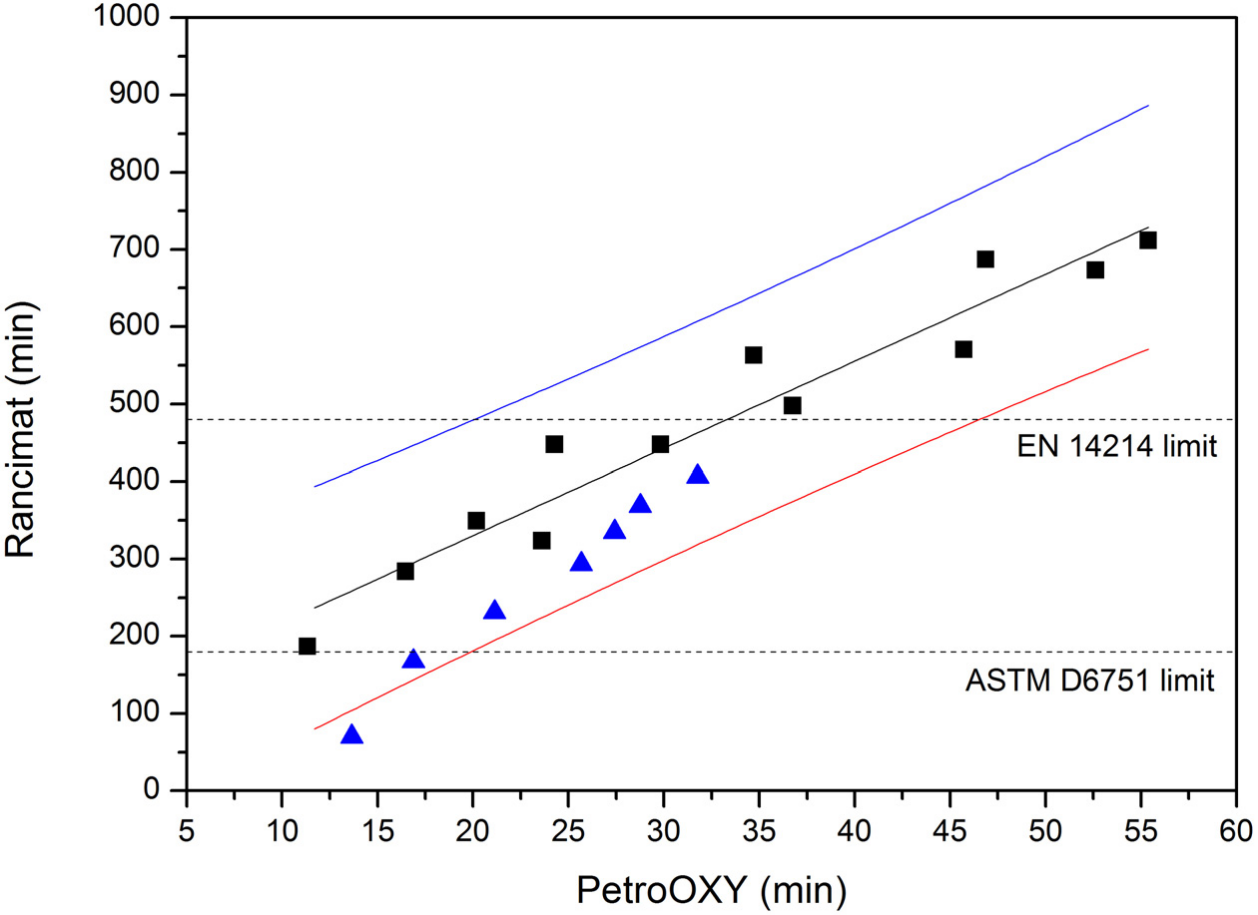
**Regression line (−), upper (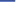), and lower (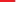) prediction intervals, experimental data (■ this work, 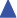 Araújo et al., 2009)**. Biodiesel with additive.

Since the data obtained with the Rancimat instrument are currently the only ones accepted by standard EN 14214, it must be noticed that, in order to use Equation 2 for estimating the Rancimat induction time from a single measurement in the PetroOXY instrument, the prediction interval must be considered. The regression line along with the prediction intervals (95% confidence) and the experimental data are shown in Figures 3, 4 for biodiesel, without and with additive, respectively.

Thus, taking into account the Rancimat threshold value of 480 min imposed by the EN 14214 standard, and given the confidence interval found around the prediction for the PetroOXY measurements, the PetroOXY time for any given sample should be higher than 26.7 min if no additive is used, and 46.6 min if an additive has been added to biodiesel. Regarding the ASTM D6751 limit, PetroOXY induction time should be higher than 17 (no additive) and 20.1 (with additive) min, in order to get a Rancimat time higher than 180 min (with the 95% confidence interval).

In Figure 4, data obtained by Araújo et al. (2009) using castor oil biodiesel and an additive (2,6-di-tertbutyl-4-methylphenol) are also shown. It can be observed that all the values but one fall within the confidence interval. It must be pointed out that castor oil biodiesel has a very different composition from the biodiesels considered in the present work, because of the high percentage of ricinoleic acid present in castor oil biodiesel (Berman et al., 2011) and that is absent in the samples used in the current study.

The results suggest that the PetroOXY method constitutes a good alternative for measuring the oxidation stability of biodiesel samples. Given its rapid determination, faster than that of the Rancimat method, and the linear correlation found between the methods, the PetroOXY instrument could be suitable for assessing biodiesel oxidation stability in future standards and norms. Nevertheless, in order to accept this method as a standard value, more data are needed, covering not only different additives and raw materials, but also to contrast the results obtained by conducting the same measurements with these instruments in different laboratories. Thus, a round robin on determining the oxidation stability using EN14112 and prEN16091 tests could be desirable.

### Influence of additives

**Figure 5:**
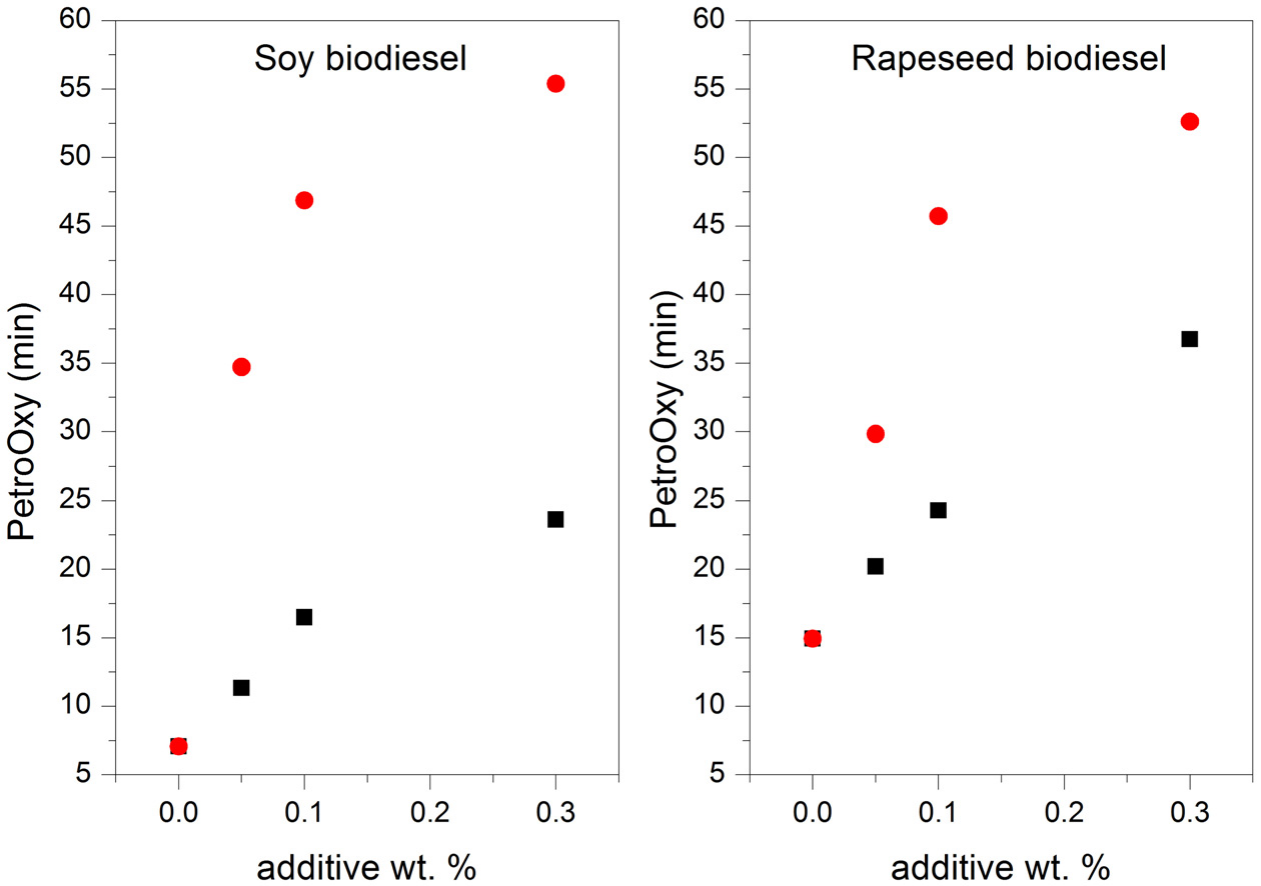
**Variation of PetroOXY induction time for soy and rapeseed biodiesel with the amount of additive used (■4-allyl-2,6-dimethoxyphenol, 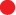 catechol)**.

The additives tested in this work exert an effect on the oxidation stability of the biodiesel samples from soy and rapeseed (Table 4). Their effect is more intense with higher percentages added to the biodiesel. Figure 5 shows the oxidation stability measurements conducted with the PetroOXY instrument vs. the concentration of additive for both biodiesel samples. The oxidation stability improved more than 400% in the case of soybean biodiesel and more than 300% in the case of rapeseed biodiesel when the highest amount of catechol was added [0.3% (m/m)]. Nevertheless, it must be borne in mind that if the added amount is too high, the use of additives could produce a negative effect on the oxidation stability of the biodiesel (Mittelbach and Schober, 2003; Domingos et al., 2007).

It can be concluded from these results that the use of catechol as a biodiesel additive yielded the best results in both cases. In addition, the use of additives clearly enhanced the oxidation stability of both biodiesel samples studied, especially in the case of soybean biodiesel.

The use of catechol as an additive blended with soybean biodiesel, even with very small amounts as low as 0.05% mass fraction, enables the restrictive limit imposed by the EN14214 standard to be complied with. However, the unblended biodiesel is far from meeting the threshold value. The addition of catechol to rapeseed biodiesel in amounts as low as 0.10% mass fraction enables compliance with the EN14214 standard.

With regards to 4-allyl-2,6-dimethoxyphenol, its use would only be satisfactory in the case of the rapeseed biodiesel blended with additive contents above 0.3% mass fraction. The rest of the blends prepared using this additive do not meet the value imposed by EN14214.

## Conclusions

An alternative method to the one accepted by standard EN14214 is used for evaluating the oxidation stability of biodiesel. The results obtained by this method, PetroOXY, show that there is a good correlation with the results obtained using the 14214 standard, the Rancimat method. The presence or not of additive in the biodiesel is a significant factor in the linear relationship between the two methods. The PetroOXY method is considerably faster. A linear correlation is proposed for determining the Rancimat oxidation stability by using the faster measurements of the PetroOXY method.

Furthermore, the use of 4-allyl-2,6-dimethoxyphenol and catechol as antioxidants for enhancing the oxidation stability of biodiesel has been proven to be very beneficial in the cases of rapeseed and soybean biodiesel, with better results for the blends with catechol. The use of catechol as an additive blended with soybean biodiesel, even in very small amounts as low as 0.05% mass fraction, enables compliance with the restrictive limit imposed by the EN14214 standard. In blends with rapeseed biodiesel, catechol contents as low as 0.10% mass fraction are sufficient to comply with the EN14214 limit.

### Conflict of interest statement

The authors declare that the research was conducted in the absence of any commercial or financial relationships that could be construed as a potential conflict of interest.
